# Implementing the WHO integrated tool to assess quality of care for mothers, newborns and children: results and lessons learnt from five districts in Malawi

**DOI:** 10.1186/s12884-017-1461-y

**Published:** 2017-08-25

**Authors:** Helen Smith, Atnafu Getachew Asfaw, Kyaw Myint Aung, Lastone Chikoti, Florence Mgawadere, Luigi d’Aquino, Nynke van den Broek

**Affiliations:** 1 Centre for Maternal and Newborn Health, Liverpool School of Tropical Medicine, Liverpool, UK; 2 UNICEF, Lilongwe, Malawi; 3 Reproductive Health Directorate, Ministry of Health, Lilongwe, Malawi

**Keywords:** Quality of care, Facility assessment, Malawi, Maternal and Newborn Health

## Abstract

**Background:**

In 2014 the World Health Organization (WHO) developed a new tool to be used to assess the quality of care for mothers, newborns and children provided at healthcare facility level. This paper reports on the feasibility of using the tool, its limitations and strengths.

**Methods:**

Across 5 districts in Malawi, 35 healthcare facilities were assessed. The WHO tool includes checklists, interviews and observation of case management by which care is assessed against agreed standards using a Likert scale (1 lowest: not meeting standard, 5 highest: compliant with standard). Descriptive statistics were used to provide summary scores for each standard. A ‘dashboard’ system was developed to display the results.

**Results:**

For maternal care three areas met standards; 1) supportive care for admitted patients (71% of healthcare facilities scored 4 or 5); 2) prevention and management of infections during pregnancy (71% scored 4 or 5); and 3) management of unsatisfactory progress of labour (84% scored 4 or 5). Availability of essential equipment and supplies was noted to be a critical barrier to achieving satisfactory standards of paediatric care (mean score; standard deviation: 2.9; SD 0.95) and child care (2.7; SD 1.1). Infection control is inadequate across all districts for maternal, newborn and paediatric care. Quality of care varies across districts with a mean (SD) score for all standards combined of 3 (SD 0.19) for the worst performing district and 4 (SD 0.27) for the best. The best performing district has an average score of 4 (SD 0.27). Hospitals had good scores for overall infrastructure, essential drugs, organisation of care and management of preterm labour. However, health centres were better at case management of HIV/AIDS patients and follow-up of sick children.

**Conclusions:**

There is a need to develop an expanded framework of standards which is inclusive of all areas of care. In addition, it is important to ensure structure, process and outcomes of health care are reflected.

## Background

It is important that care for mothers, newborns and children is both available and of good quality. This is reflected in the latest global initiatives such as the strategies towards ending preventable maternal mortality (EPMM), the Every Newborn Action Plan (ENAP), and the new Global Strategy for Women’s, Children’s and Adolescents’ Health (2016–2030) for the post-2015 Sustainable Development Goal era [1–3]. Leaders of global health agencies have agreed an agenda for better measurement of the quality of healthcare and aim to align the various efforts, reduce the burden of data collection and reporting for countries and improve linkage of results with decision-making. New tools for the assessment of quality have been developed and international consensus reached on indicators for quality of care in maternal, newborn and child health [4, 5].

In 2014 the World Health Organization (WHO) developed a new integrated tool to assess the quality of care, designed to help the Ministry of Health (MoH), key stakeholders and partners in maternal, newborn and child health (MNCH) to carry out comprehensive assessments at facility level. The objectives, structure and methods differ from other global facility assessment tools currently in use in that it allows for an assessment of the quality of care provided, not just the quantity or availability. For example, the World Bank Service Delivery Indicators (SDI) initiative collects evidence on the quantity of health services to help decision makers track progress and to benchmark countries [6], and the WHO Service Availability and Readiness Assessment (SARA) monitors tracer indicators of service availability and readiness, with a focus on provision of interventions across the continuum of care, in order to support health system strengthening [7]. However, despite their related focus, neither of these tools assess the quality of care. The new WHO integrated tool in principle, enables an assessment of the quality of care provided against national standards (accepted and established in context) to produce an overall diagnosis of and to identify obstacles to quality of care. It is designed to be both a management and an evaluation tool. It is proposed that this tool is used country-wide as a component of a quality improvement strategy, or in a representative sample of healthcare facilities. It can potentially also be used in a single health facility to track progress in quality of care and inform quality improvement activities.

The new WHO tool for assessment of Quality of Care incorporates existing survey modules and instruments, namely the Health Facility Survey to evaluate quality of care for sick children [8], and the quality assessment and improvement tool for hospital care for mothers and newborn babies [9]. The new fully integrated tool was used for the first time in Malawi in 2015.This study reports on the practical feasibility of using the tool, its limitations and strengths. We present the key findings of the assessment as well as recommendations for adaptation and implementation of the tool at scale.

## Methods

### Adaptation and familiarization with the tool

In Malawi, WHO and MoH staff reviewed and adapted the WHO tool for assessment of quality of care to the epidemiology and health system context of the country. A team of national and international assessors comprising senior clinicians, nurses, representatives from medical and nursing training institutions, professional associations, committees in charge of national treatment guidelines and practicing doctors and nurses reviewed the tool and agreed the standards to be included. Standards were derived from the Malawi Standard Treatment Guidelines [10], the National Integrated Maternal and Newborn Health Management guidelines and the WHO Pocket Book of Hospital Care for Children [11]. The tool was then pilot-tested in two hospitals (not included in the assessment) for the assessors to become familiar with the instrument and assess time needed to collect data.

### Structure of the tool

The WHO tool for assessment of quality of care comprises four modules related to A) infrastructure, B) maternal, C) newborn and D) paediatric care. In Malawi two versions of the tool were used; one for district hospitals or tertiary care centres designated to provide Comprehensive Emergency Obstetric and Neonatal Care (CEmOC) and one for use at health centre level or health facilities designated to provide Basic Emergency Obstetric and Neonatal Care (BEmOC). The only difference between the two tools is that the CEmOC tool has three additional areas that are assessed: i) case management of Caesarean section, ii) paediatric inpatient care and iii) paediatric surgery and rehabilitation. Data was collected via: pre-formatted questionnaires; checklists for availability of services, equipment, drugs and supplies; structured forms for scoring of case management observations against standards of care; and exit interviews for health service providers, caretakers or mothers. All assessors were trained and familiarised with national standards of care across the four modules (three days). Practical sessions were held at a health facility to practice the use of the tool and scoring method.

Module A gathers basic information about infrastructure, ward layout and organisation of care including staffing. Modules B, C and D assess quality of maternal, neonatal and child health care respectively and each includes sections on: emergency care, in patient care, infection control and supportive care, essential drugs, equipment and supplies, case management, and monitoring and follow up. In total the CEmOC tool assesses 45 variables (10 variables relating to infrastructure, 14 for maternal care, 9 for neonatal and 13 for paediatric care) and the BEmOC tool assesses 43 variables (10 infrastructure, 13 maternal care, 9 neonatal care, and 11 paediatric care).

### Scoring of each component of care

Across all four modules each aspect of care is observed and then scored 1–5 (5: good practice complying with standard of care; 4: little need for improvement to reach standard of care; 3: some need for improvement to reach standard of care; 2: considerable need for improvement; and 1: services are not provided, there is totally inadequate care or potentially life-threatening practice). For each area of care assessed, several standards and components of care are scored. For example, for emergency obstetric care standards relating to patient flow and layout and structure of emergency care are individually scored. The section ends with a summary table which includes a summary score (an average of all the standards assessed) in that section and space to document main strengths and weaknesses (in writing).

### Study sites

The selection of the five districts for inclusion in this assessment was opportunistic; they are districts where the Centre for Maternal and Newborn Health (CMNH) has an ongoing programme of work to improve availability and quality of Emergency Obstetric Care and early Newborn Care. Nevertheless, they were considered representative of the 28 districts and three regions of Malawi; two target districts are in Central region, two in Southern region, and one in Northern region. Within each district each healthcare facility providing CEmOC (i.e. the district hospital) and the five largest (according to number of births per year) healthcare facilities providing BEmOC were included. Thirty-three healthcare facilities are under the direct control of the Malawi MoH, and two healthcare facilities in district 5 are members of the Christian Health Association of Malawi. Healthcare facilities were assessed using either the CEmOC or BEmOC assessment tool depending on the level of care provided; in each district, at least one CEmOC and five BEmOC facilities were assessed. In total six CEmOC and 29 BEmOC assessments were carried out.

### The assessment process

Assessments took between one to three days depending on the type and size of healthcare facility. WHO and the MoH trained district level teams to carry out facility visits; teams were multidisciplinary and included district health officers, midwives, nurses, obstetricians, paediatricians and general physicians with enough experience to make valid observations of the care provided. Most healthcare facilities were assessed by a wholly ‘external’ team, except for district hospitals where teams included one ‘internal’ staff member; typically, teams included three or four people. All facility assessments were conducted between July–August 2015.

### Data analysis

**Fig. 1:**
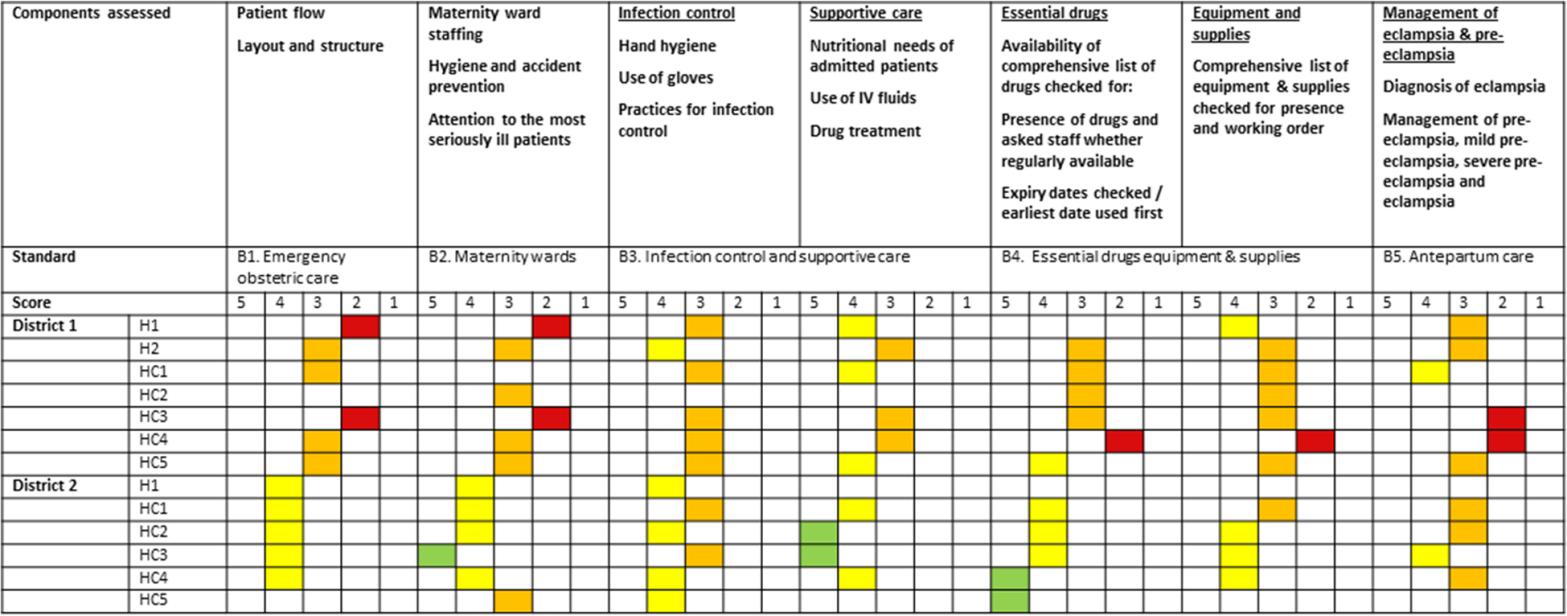
Summary scores by standard for each healthcare facility: maternal care example

The MoH and WHO developed a preliminary analysis plan, which CMNH adapted and used for aggregating data and reporting across the five districts. A Likert scale (1–5) was used to assess degree of compliance with standards of care. Basic descriptive statistics were used to analyse summary scores for each standard (proportion of healthcare facilities where standards were met or not) and measures of central tendency (mean scores, standard deviation (SD)). Data were entered manually and analysis was done using Excel 2013. To help districts and healthcare facilities readily identify areas in need of improvement, we developed a ‘dashboard’ to display summary scores for each standard, by facility (Fig. 1). The figure displays summary scores only; these represent the average score across all components assessed for each area of care.

## Results

Data was obtained from 31 of a total of 35 healthcare facilities assessed; two facility assessments in districts 4 and 5 were not received by CMNH and paper files were reported as missing. Data for each module are summarised in Table 1 as proportion of healthcare facilities where standards are met in each district. The mean score and standard deviation for each standard assessed are presented across all five districts, as well as for the different levels of care (CEmOC and BEmOC).

**Table 1:** Key findings and summary scores by module

Area of care	Standards assessed	Number (n) and proportion (%) of facilities where standards are met (scoring 4 or 5)						Mean score	Mean score	
		District 1 *n* = 7 (%)	District 2 *n* = 7 (%)	District 3 *n* = 7 (%)	District 4 *n* = 5(%)	District 5 *n* = 5(%)	Total *N* = 31 (%)	Across 5 districts (SD)	Level of care	
Module A: Infrastructure									CEmOC	BEmOC
A2. Infrastructure	Availability of electricity, back-up power supply, running water, soap/disinfectant, sharps disposal, fridge for drugs, oxygen source, complaints/ suggestions box	3 (42.8)	5 (71.4)	4 (57.1)	3 (60)	nd^a^	15 (48.3)	3.8 (0.83)	4.3 (0.6)	3.8 (0.85)
A5. Health information & record keeping	Health info: reporting forms, monthly reports of OPD visits, computer based system for medical indicators, periodic facility review of stats and indicators Records: clarity, legibility, availability, access	2 (28.6)	5 (71.4)	4 (57.1)	2 (40)	nd	13 (41.9)	3.8 (0.79)	4.0 (0.8)	3.7 (0.81)
A6. Essential drugs & blood products	List of essential drugs checked for presence, regular availability, expiry Blood bank at facility, blood units available	2 (28.6)	6 (85.7)	1 (14.3)	3 (60)	2 (40)	14 (45.2)	3.8 (0.73)	4.3 (0.5)	3.7 (0.75)
A7. Laboratory	Various lab investigations observed: if test available, results readily available, average time to get results	3 (42.8)	3 (42.8)	2 (28.6)	4 (80)	nd	12 (38.7)	3.3 (1.06)	4.2 (0.4)	3.1 (1.06)
A8. Health facility policies	Policies checked for availability: hospital fees, free drugs, services for emergency cases, community participation, infection control, hand hygiene, in service training, quality of care, KMC, essential drugs list	2 (28.6)	2 (28.6)	5 (71.4)	4 (80)	nd	13 (41.9)	3.5 (0.74)	4.0 (0.0)	3.3 (0.79)
A9. Referral	Functioning vehicle with fuel routinely available for emergency referral use, referral transport provided free of charge, clear criteria for referral, communication with referral facility, referral time	1 (14.3)	7 (100)	4 (57.1)	4 (80)	nd	16 (51.6)	3.7 (1.13)	4.4 (0.9)	3.6 (1.15)
A10. Guidelines/ audit	Guideline availability for normal labour & delivery, emergency conditions for mothers, newborns and children, routine care for newborns, management of childhood illnesses, recent obstetric, neonatal and paediatric textbook Conduct of audits to review deaths and complications (MDSR, neonatal, perinatal, paediatric), functioning of teams, recommendations discussed and implemented, periodic meetings to discuss issues and revise protocols	0 (0)	3 (42.8)	2 (28.6)	0 (0)	nd	5 (16.1)	2.6 (1.11)	3.5 (0.6)	2.4 (1.12)
Module B: Maternal Care									CEmOC	BEmOC
B1. Emergency obstetric care	Patient flow, layout and structure of emergency care	0 (0)	6 (85.7)	3 (42.9)	3 (60)	1 (20)	13 (41.9)	3.4 (0.77)	3.2 (0.8)	3.5 (0.79)
B2. Maternity wards	Ward staffing, hygiene & accident prevention, attention to most seriously ill	0 (0)	6 (85.7)	2 (28.6)	2 (40)	1 (20)	11 (35.4)	3.2 (0.77)	3.0 (0.9)	3.3 (0.75)
B3. Infection control & supportive cares	Infection control: hand hygiene, use of gloves, infection control practices	1 (14.3)	5 (71.4)	0 (0)	0 (0)	0 (0)	6 (19.3)	3 (0.74)	3.2 (0.8)	3.0 (0.75)
	Supportive care: nutritional needs of admitted patients, IV fluids, drug treatment, blood transfusion	3 (42.9)	5 (71.4)	4 (57.1)	5 (100)	5 (100)	22 (70.9)	4 (0.62)	3.8 (0.4)	4.1 (0.65)
B4. Essential drugs, equipment and supplies	Essential drugs: checked for presence, regular availability, expiry: water, normal saline. Ringers lactate, hydralazine/nifedipine, methyldopa, oxytocin, misoprostol, ergometrine, calcium gluconate, injectable MgSO4, diazepam, ampicillin/amoxycilin, benzylpenicillin, gentamycin, metronidazole, lignocaine 2% or 1%, halothane/isoflurane, ketamine	1 (14.3)	6 (85.7)	1 (14.3)	2 (40)	2 (40)	12 (38.7)	3.5 (0.87)	4.0 (1.0)	3.4 (0.85)
	Equipment and supplies: checked for presence, safety, hygiene, good working order: lighting, exam light, wall clock, delivery pack, partographs, heat lamp, CS set, oxygen cylinder, concentrator, supply & flow metres and equipment for administration of oxygen, respiratory support bags, bags and masks (adult and neonatal size), anaesthetic equipment, thermometer, sterile gloves, sterile gauze, foetal stethoscope, stethoscope, BP machine, infusion sets & pumps, IV catheters, urinary catheter & bags, syringes, needles & cannulas, suture set, sharps container, vacuum extractor, vacuum aspirator, delivery, regular & operating beds, neonatal equipment	1 (14.3)	4 (57.1)	3 (42.9)	4 (80)	3 (60)	15 (48.4)	3.4 (0.73)	3.8 (0.4)	3.4 (0.77)
B5. Antepartum care	Eclampsia and pre-eclampsia: diagnosis, management of mild pre-eclampsia, management of severe pre-eclampsia and eclampsia	1 (14.3)	2 (28.6)	4 (57.1)	5 (100)	0 (0)	12 (38.7)	3.3 (0.77)	3.4 (0.5)	3.3 (0.82)
	Infections during pregnancy: Antibiotic use inf pregnancy, screening and management of HIV in pregnancy, management of severe malaria in pregnancy	5 (71.4)	4 (57.1)	3 (42.9)	5 (100)	5 (100)	22 (71)	4 (0.54)	4.2 (0.4)	4.0 (0.58)
B5. Intrapartum & postpartum care	Normal labour and vaginal delivery: delivery conditions, supportive care throughout labour, care during first, second and third stage of labour, care of mother after delivery	1 (14.3)	5 (71.4)	4 (57.1)	1 (20)	2 (40)	13 (41.9)	3.5 (0.70)	3.0 (0.0)	3.6 (0.73)
	Preterm labour: management of pre-term labour, antenatal administration of corticosteroids	2 (28.6)	3 (42.9)	1 (14.3)	1 (20)	4 (80)	11 (35.5)	3 (1.33)	4.3 (0.5)	2.6 (1.23)
	Caesarean section (Assessed at 6 EmONC facilities only): emergency CS, timing of CS and informed consent, indications and policies to reduce inappropriate CS, procedures related to CS, immediate post-CS care, care after 24 h and discharge after CS	1 (50)	nd	1 (100)	1 (100)	1 (100)	4 (66.6)	3.6 (0.89)	3.5 (1.0)	nd
	PPH guidelines available and known by SBAs, adequate equipment, drugs and personnel available, uterine tonus controlled after delivery, reliable way of measuring blood loss in delivery room and early puerperium, blood can be obtained without delay 24 h a day, written protocols available, known and used by staff for notification of on calls, treatment and resuscitation, uterine atony, and refractory haemorrhages after treatment	0 (0)	7 (100)	1 (14.3)	3 (60)	3 (60)	14 (45.2)	3.5 (0.97)	3.4 (0.5)	3.6 (1.06)
	Unsatisfactory progress of labour: diagnosis of labour disorders, prolonged active phase management	6 (85.7)	7 (100)	3 (42.9)	5 (100)	5 (100)	26 (83.9)	4.2 (0.57)	4.2 (0.4)	4.2 (0.61)
	Monitoring & follow up of admitted patients: monitoring of individual progress, reassessment by midwives, reassessment by clinicians, follow up	1 (14.3)	6 (85.7)	3 (42.9)	4 (80)	3 (60)	17 (54.8)	3.6 (1.0)	3.5 (0.8)	3.6 (1.09)
Module C: Neonatal Care									CEmOC	BEmOC
C1. Nursery facilities	Ward staffing, hygiene & accident prevention	0 (0)	4 (57.1)	5 (71.4)	3 (60)	1 (20)	13 (41.9)	3.2 (1.19)	3.5 (0.8)	3.1 (1.3)
C2. Infection control & supportive care	Infection control and hygiene, use of gloves, infection control practices	2 (28.6)	4 (57.1)	3 (42.9)	0 (0)	0 (0)	9 (29)	3.3 (0.93)	3.2 (0.4)	3.6 (1.06)
	Supportive care: IV fluids use, drug treatment, central venous cannulation & umbilical catheterization, blood transfusion	0 (0)	2 (28.6)	5 (71.4)	2 (40)	0 (0)	9 (29)	3.5 (0.83)	3.7 (1.2)	3.5 (0.65)
C3. Essential drugs, equipment & supplies	Essential drugs checked for presence, regular availability, expiry: antibiotics, antivirals and antifungals, CNS drugs, respiratory drugs, miscellaneous including chlorhexidine, paracetamol suspension, nevirapine suspension, vitamins & minerals	3 (42.9)	3 (42.9)	5 (71.4)	2 (40)	1 (20)	14 (45.2)	3.4 (1.0)	4.0 (0.0)	3.2 (1.17)
	Equipment and supplies checked for presence, safety, hygiene, good working order: incubators, radiant warmers, baby cots, phototherapy lamps, ambu bag, oxygen supply, concentrator & nasal prongs, appropriate sized face masks, CPAP systems, multifunction monitors, pulse-oximeters, breast pumps, nasogastric tubes, microdroppers, exchange transfusion, stethoscope, glucometers and sticks, suction apparatus, thermometers, electric weighing scale, linen	1 (14.3)	2 (28.6)	2 (28.6)	2 (40)	0 (0)	7 (22.6)	2.9 (0.95)	3.3 (0.8)	2.8 (0.98)
C4. Routine care	Neonatal resuscitation, assessment & immediate care, screening, prevention & management of infectious diseases, early & exclusive breastfeeding, monitoring before discharge, counselling for mothers	1 (14.3)	2 (28.6)	7 (100)	5 (100)	0 (0)	15 (48.4)	3.7 (0.70)	3.5 (1.0)	3.81 (0.54)
C5. Management of sick newborn	Management of preterm and LBW babies, neonatal sepsis, recognition & treatment of jaundice, management of convulsions, feeding for sick neonates	0 (0)	1 (14.3)	3 (42.9)	1 (14.9)	0 (0)	5 (16.1)	3.2 (0.93)	3.2 (1.2)	3.2 (0.75)
Module D: Paediatric Care									CEmOC	BEmOC
D1. Emergency paediatric care	Patient flow, emergency care staffing, layout & structure of emergency care, case management of emergency conditions	0 (0)	4 (57.1)	1 (14.9)	0 (0)	3 (60)	8 (25.8)	3.2 (0.69)	3.4 (0.9)	3.1 (0.64)
D2. Paediatric wards	Staffing level, hygiene & accident prevention, attention to most seriously ill patients	0 (0)	1 (100)	1 (100)	nd	nd	2 (33.3)	3.2 (0.96)	3.3 (1.0)	nd
D3. Infection control & supportive care	Infection control: Hand hygiene, use of gloves, practices for infection control	0 (0)	0 (0)	5 (71.4)	0 (0)	3 (60)	8 (25.8)	3.2 (0.60)	3.4 (0.5)	3.2 (0.62)
	Supportive care: Nutritional needs, use of IV fluids, drug treatment, blood transfusion	5 (71.4)	4 (57.1)	7 (100)	4 (80)	2 (40)	22 (71)	4.2 (0.66)	2.6 (0.9)	2.8 (1.11)
D4. Essential drugs, equipment & supplies	Essential drugs checked for presence, regular availability, expiry: glucose (30–50%, 10%, 5%), saline, ringers lactate, half strength darrows, RESOMOL, adrenaline, corticosteroids IV or oral, furosemide IV, first line anticonvulsant, antibiotics, anti-malarials, paracetamol (oral, suppository), vitamin K, ORS, zinc sulphate	3 (42.9)	3 (42.9)	3 (42.9)	2 (40)	3 (60)	11 (35.5)	3.5 (0.76)	3.4 (0.5)	3.5 (0.81)
	Equipment and supplies checked for presence, safety, hygiene, good working order: resuscitation table, torch, autoscope, scales for children, measuring board, stethoscope, thermometer, heat source, oxygen cylinder, concentrator & supply, oxygen flow meter, supplies for oxygen administration, resuscitation bags, IV sets, infusion pumps, burettes, cannulas/butterflies paediatric size, feeding syringes, NG tubes	0 (0)	1 (14.9)	2 (28.6)	0 (0)	2 (40)	5 (16.1)	2.7 (1.1)	2.6 (0.9)	2.8 (1.11)
D5. Case management of common diseases	Cough/difficult breathing: assessment & treatment of pneumonia, administration of antibiotics, administration of oxygen therapy, use of chest x-ray, management of wheezing, TB treatment	1 (14.9)	1 (14.9)	2 (28.6)	4 (80)	2 (40)	10 (32.3)	3.4 (0.91)	3.2 (0.4)	3.5 (1.0)
	Diarrhoea: assessment of dehydration, management of dehydration & diarrhoea, antibiotic use, continued feeding	3 (42.9)	2 (28.6)	5 (71.4)	4 (80)	3 (60)	17 (54.8)	3.7 (0.68)	3.4 (0.5)	3.8 (0.7)
	Fever: differential diagnosis & investigations, diagnosis & management of meningitis, diagnosis & management of severe/complicated malaria, diagnosis & management of measles, diagnosis & management of other severe febrile conditions	1 (14.9)	3 (42.9)	7 (100)	4 (80)	4 (80)	19 (61.3)	3.8 (0.62)	3.6 (0.5)	3.9 (0.64)
	Malnutrition: assessment of nutritional status, management of infection & micronutrient supplementation, management of dehydration, prevention & management of hypoglycaemia & hypothermia, feeding of severely malnourished children, management of associated conditions & supportive care	4 (57.1)	0 (0)	6 (85.7)	4 (80)	4 (80)	18 (58)	4 (0.59)	3.8 (0.4)	4.0 (0.63)
	HIV/AIDS: counselling & diagnosis of paediatric HIV/AIDS, ARV treatment and monitoring, opportunistic infections & supportive care, supportive care & follow up of HIV infected children	3 (42.9)	3 (42.9)	6 (85.7)	4 (80)	3 (60)	19 (61.3)	4.3 (0.66)	3.7 (0.6)	4.4 (0.62)
D5. Monitoring & follow up of admitted children	Monitoring of individual progress, monitoring by nurses, reassessment by clinicians, follow up	1 (14.9)	0 (0)	6 (85.7)	2 (40)	2 (40)	11 (35.5)	3.8 (1.0)	2.6 (0.5)	4.2 (0.86)
D6. Paediatric surgery & rehabilitation	Paediatric size anaesthesia-equipment, pre-, intra- and post-operative care, rehabilitation	nd	nd	nd	nd	0 (0)	1 (16.7)	3.5 (0.70)	3.5 (0.7)	nd

^a^ *nd* no data

Across all five districts, referral is the only aspect of facility infrastructure/organisation of care where more than half of all healthcare facilities (51.6%) meet the standard or require little improvement (mean score: 3.7; SD 1.13) Other aspects of infrastructure and organisation that scored well across the five districts were infrastructure (3.8; SD 0.83), availability of essential drugs and blood products (3.8; SD 0.73), and health information systems (3.8; SD 0.79). Healthcare facilities scored less well in relation to standards for laboratory systems (3.3 SD 1.06) and standards relating to availability of guidelines and conducting audit or review (2.6; SD 1.11).

Three areas of maternal care met standards or required little improvement: supportive care (71% of healthcare facilities scored 4 or 5); prevention and management of infections during pregnancy (71% of healthcare facilities scored 4 or 5); and management of unsatisfactory progress of labour (84% of healthcare facilities scored 4 or 5). Average scores were lowest for infection control (3; SD 0.74), maternity wards (3.2; SD 0.77) and prevention and management of preterm labour (3; SD 1.33).

Fewer than half of all healthcare facilities comply with standards or require little improvement for neonatal care. Fifteen healthcare facilities (48% of the total) score 4 or 5 for routine neonatal care. For all other standards assessed the average score was 3.5 or less with problems highlighted for essential equipment and supplies (2.9; SD 0.95), nursery facilities (3.2; SD 1.19), and management of the sick newborn (3.2; SD 0.93).

Across the five districts, healthcare facilities generally scored well in relation to supportive care for sick children (4.2; SD 0.66); management of HIV/AIDS cases (4.3; SD 0.66) and management of malnutrition (4; SD 0.59). However, availability of essential paediatric equipment and supplies (2.7; SD 1.1), emergency paediatric care (3.2; SD 0.69), and infection control (3.2; SD 0.60) are areas in need of improvement.

### Results by level of care

Table 1 presents mean scores for the standards disaggregated by level of care (CEmOC and BEmOC). Overall mean scores for all aspects of facility infrastructure and organisation of care are higher for CEmOC facilities. CEmOC and BEmOC facilities have similar average scores for most aspects of maternal care, although for availability of essential drugs, and management of preterm labour, the mean score is higher at CEmOC facilities. For neonatal care average scores are similar across the levels of care for most standards, except availability of essential drugs, equipment and supplies where CEmOC facilities have a higher mean score. For the paediatric care standards, there are no differences in average scores between CEmOC and BEmOC facilities, except for case management of HIV/AIDS patients and monitoring and follow-up of admitted children where BEmOC facilities have higher average scores.

### Key emerging issues

Healthcare facilities in district 1 presented the most gaps and challenges, with an average score of all assessed standards of 3 (SD 0.19) across all healthcare facilities in the district. The healthcare facilities assessed in district 2 emerged as the best performing with an average score of all assessed standards of 4 (SD 0.27) across all healthcare facilities. The availability of essential equipment and supplies remains a critical barrier to achieve satisfactory standards of care. In particular, the availability of equipment and supplies for both neonatal care (2.9; SD 0.95) and child care (2.7; SD 1.1) was deemed to be insufficient. Infection control was also a cross-cutting area in all districts that emerged as a barrier to quality of care provided along the continuum of care, with an average score of 3 (SD 0.74) for maternal care, 3.3 (SD 0.93) for newborn care and 3.2 (SD 0.60) for paediatric care.

Case management along the continuum of care was generally scored above 3.5 across all districts and healthcare facilities, however, there were some critical emerging areas. For maternal care, standards for maternity wards (3.2; SD 0.77) and prevention and management of preterm labour (3; SD 1.33) required improvement. For newborn care, management of the sick newborn (3.2; SD 0.93); nursery facilities (3.2; SD 1.19) and emergency paediatric care (3.2; SD 0.69) require further improvement.

## Discussion

This is the first time the WHO integrated tool has been used to assess quality of maternal newborn and child health care at country level. Application of the tool is feasible and has provided valuable information highlighting areas of good quality care as well as where there are deficiencies in the quality of care at healthcare facility and district levels in Malawi. Using a “dashboard” to display the assessment findings makes it possible to easily identify priority areas of care that require immediate action. This study also highlights the need for modification and further standardisation of the new WHO Quality of Care tool. In particular, we recommend a reduction in the overall number of standards assessed, revision of the current set of standards such that all aspects of the continuum of care are included and revision of the formulation of standards such that these are specifically reflective of all aspects of quality (i.e. both with regard to inputs, process and outcome), measurable and adaptable to context (healthcare facility level).

### Strengths and limitations of the new WHO tool

While the integrated tool is designed to assess quality across the continuum of care, the standards currently included in the tool are not fully representative of all the areas of care that need to be assessed. Antenatal care is not assessed at all and postnatal care in a very limited way. These are typically neglected areas of care which are often not included in quality improvement activities. This is in part because national standards for antenatal and postnatal care are often not in place. Developing such standards and including them in comprehensive quality of care assessments is a priority. In addition, the tool would be enhanced by including indicators for routine intrapartum care practices, for example the choice of a companion at the time of birth, freedom in position and movement throughout labour, non-supine position in labour and careful monitoring of progress with the partograph [12]. These aspects of care, together with others relating to women’s experience of care (e.g. effective communication, care with respect and dignity), are essential and inter-linked dimensions of quality yet are difficult to assess and monitor well. Methods such as structured observation and properly conducted exit interviews with women would be appropriate to measure these aspects of care and could easily be incorporated into a revised version of the tool.

There are some important points to highlight in relation to how well the tool was able to provide complete and accurate data. It proved difficult to report on the size and capacity of the healthcare facilities assessed as the data on basic hospital statistics and outcome measures were often not available and were not collected consistently. In addition, for neonatal and paediatric module, data collection was frequently incomplete. In its current format, the tool is very long and detailed.

Some standards are easier to assess (e.g. for ward infrastructure) than others (e.g. for satisfactory progress in labour), some are better defined (e.g. criteria for the standard on referral) than others (e.g. availability of essential drugs, equipment and supplies). This does make it more likely that some aspects of care are scored more highly than others simply because the relevant “standard” is easier to measure and more accurately defined. For example, it would be more accurate and informative to collect data on stock-outs or non-availability of specific essential drugs.

Completion of exit interviews with women, caretakers and providers is a mandatory component and while some healthcare facilities did complete these, we did not have access to the complete data set and so have not reported these findings. Where case observation is used, it is not clear how many cases assessors should observe before judging whether standards are met or not. There are also challenges to relying on observation; especially the potential for assessor bias and the likely impact on provider behaviour of having an external assessor present [13]. Peer or self-assessment at healthcare facilities are alternative approaches. In addition, if there are no cases available to observe at the time of the assessment this part of the assessment cannot be completed. In these circumstances, assessors are advised to use staff interviews and data from registers to gather relevant information where possible.

Data collection could be made more efficient via use of technology including tablets or machine readable forms, and this is something to consider for future iterations of the tool. Table 2 summarises our key recommendations for improvement of the integrated tool.

**Table 2:** Recommendations for improvement of the WHO integrated quality of care assessment tool

Shorten the tool	Because it is so comprehensive, the tool can be adapted to suit local disease and population profiles and used to assess different levels of care. However, the adapted assessment tool applied in Malawi is over 200 pages long. A shorter tool, focused on priority areas of care, may be more appropriate for use in busy clinical environments.
Combine the CEmOC and BEmOC tools	There are currently two versions of the tool and the only difference is that the CEmOC tool assesses management of Caesarean section (including pre-, intra- and post-operative procedures), paediatric wards and paediatric surgery and rehabilitation. It should be possible to combine the tools into one, with separate modules for assessing the standards relevant to CEmOC facilities only.
Streamline data sources and revise instructions for data collection	The data sources could be streamlined, and clearer instructions for conducting the various components of the assessment provided. The number of data collection methods and lack of clear guidance on when and how they should be used is potentially confusing to assessors who have limited time to complete facility visits.
Simplify the assessment of availability of drugs and equipment	The assessment of drugs, equipment and supplies could be simplified for example by focusing on fewer ‘tracer’ essential drugs and obtaining information on stock outs or unavailability rather than amount and quality of drugs.
Link the assessment tool to existing quality of care frameworks and indicators	It needs to be made clear how the tool and the components assessed map onto the new quality of care framework and indicators being developed by WHO as well as other maternal and newborn health indicators being developed to measure quality of care at facility level.
Consider alternatives to external assessors	Given the potential for assessor bias and for the presence of an external assessor to produce changes in provider behaviour (the Hawthorn or observer effect), the tool may produce more valid data if completed by staff working at the facilities.
Revise the action planning process	An important drawback for practical use of the assessment data is that facility action plans were not completed It may be that this exercise is best coordinated at district level, where common problems with quality of care can be identified and resources targeted towards supporting facilities to make improvements. Staff at individual facilities may become overwhelmed if they try to tackle all the areas of care identified as in need of improvement. The colour coded dashboard (Fig. 1) may be a useful template for displaying data and could be used as a basis for discussion and prioritisation of problems for immediate and longer term action.

We have not reported on the resource requirements for implementing the WHO integrated quality of care assessment tool at national or sub-national level. These data could be generated reliably in future through careful implementation research conducted alongside country level assessments. The burden of collecting a large number of (additional) data on quality of care and performance at scale is a factor that other pilot assessments have encountered [14] and for this reason we would recommend that the tool is shortened whenever possible and/or that selected components or modules are used as needed.

The debriefing and action plan provided in the assessment tool annex was not completed for any healthcare facility, and the reasons for non-completion of this critical step in the process need to be understood, perhaps through dialogue with assessors.

### Implications for policy and practice

Until recently, the emphasis has been on coverage and availability of care rather than quality [15]. A new tool to measure quality is, in principle, useful to provide baseline information and highlights specific areas for quality improvement. The new WHO tool has this potential. A key bottleneck in quality improvement efforts at healthcare facility level in Malawi and other low- and middle-income countries is translation of assessment data to action. The dashboard approach highlights in a very visual and accessible way where the key quality of care problems exist at both healthcare facility and district level. The findings were presented at a national workshop to share lessons learnt on maternal, newborn and child health quality of care in Lilongwe, where the Minister for Health in Malawi recommended that a dashboard similar to the one developed for this analysis be adopted to help map quality of care at district level. Subsequently, the assessment data were disseminated at district level and action plans were developed. A similar standards-based action-oriented healthcare facility assessment approach has been implemented in other lower-middle income countries [16, 17], and is the core component of clinical or standards-based audit [18].

At a global level, the shift towards improving quality of maternal and newborn health services demands a coordinated approach. Yet measurements of quality of care are often not done consistently and there are many different tools, indicators and methods, making it difficult to compare between and within countries. There is a need to clarify where and how the new integrated WHO tool fits with other facility-based assessment tools, such as SARA and the World Bank Service Delivery Indicators (SDI) survey. The new WHO Quality of Care tool is unique in its ability to judge quality not just quantity of services, but it assesses relatively more structure and process characteristics; ideally a healthcare facility assessment tool should assess quality in relation to structure, process and outcome [19]. There are plans to extend the SARA assessment to include structured observations of consultations between providers and women for, as well as vignettes to determine providers’ usual case management practices [20]. It is essential that partners prioritise alignment of quality of care assessment tools. The recent development and testing by WHO and partners of a core set of harmonized maternal newborn and child health indicators is a step in the right direction, but the final indicators need to be rapidly integrated into existing tools [4]. In addition, as with any new tool developed by international agencies, it is imperative that the standards on which the tools are based are accepted by healthcare workers and established in the local context as realistic. A recent assessment of quality of care in a low resource referral hospital in Zanzibar used a participatory approach with skilled birth attendants and midwifery and obstetrics specialists to agree realistic criteria for quality of care that reflected local realities [21]. In this ‘bottom-up’ approach fetal heart rate assessment every 30 min was maintained as ‘optimal’ practice, but team agreed that assessments within intervals of 90 min were an acceptable audit criterion.

## Conclusion

Facility-based assessment methods are often time consuming and expensive to use at scale. The new WHO integrated quality of care assessment tool in its current format contains a number of separate modules even though not all areas of care are represented yet. The data produced requires interpretation and discussion to distil practical advice on improvements to care. This assessment identified important lessons for future development of the tool, including shortening it and/or using specific modules at a time, streamlining data collection methods and data sources and revision of standards to ensure the three components (inputs, process and outcomes) of quality are included. With modification, the tool could be used in other countries for baseline and subsequent periodic assessments of the quality of care.

## Abbreviations

BEmOC: Basic Emergency Obstetric and Neonatal Care
CEmOC: Comprehensive Emergency Obstetric and Neonatal Care
CMNH: Centre for Maternal and Newborn Health
ENAP: Every Newborn Action Plan
EPMM: Ending preventable maternal mortality
MNCH: Maternal, newborn and child health
MoH: Ministry of Health
SARA: Service Availability and Readiness Assessment
SDI: Service Delivery Indicators
WHO: World Health Organization
